# Less care today, more tomorrow? A study of the impact of financial hurdles in access to care on future health status and health consumption

**DOI:** 10.1186/1475-9276-11-S1-A3

**Published:** 2012-01-23

**Authors:** Paul Dourgnon, Romain Fantin, Florence Jusot

**Affiliations:** 1 Institut de Recherche Documentation en Economie de la Santé (IRDES), Paris, France; 2 Laboratoire d’économie et de gestion des organisations de santé (LEGOS), Paris, France

## Context

Several studies provide evidence of horizontal inequities in health care use in France, i.e. differences in health care utilization for equal needs in favor of the highest socioeconomic groups [1-3]. Similarly, significant social inequalities in mortality have been found in France in comparison with other European countries [4]. However, the contribution of inequity in access to care to health inequalities is disputable and the consequences in term of health status and future health care consumption of horizontal inequities in health care use have scarcely been explored.

## Material and methods

We explore relationships between self assessed unmet needs for financial reasons and 1/ future health status 2/ future health care consumption. We base our analysis on individual data from the French panel Health, Health Care and Insurance Survey (ESPS). The pooled sample contains 8 000 individuals observed twice with a 4 years interval, among which 16% reported unmet needs.

In addition to questions on self-assessed health status and socioeconomic characteristics respondents assessed unmet needs for financial reasons within the last twelve months. The survey data is merged with administrative data from social sickness funds, providing exhaustive information on health consumption during each period.

We use an econometric model to identify longitudinal relationships between past unmet needs and future health status and health consumption. The first model addresses evolution of health status between first and second observations (year1 and year2) and how it can be explained by unmet needs in year1, when controlled by socioeconomic status, age and gender. The second model explains health care utilization in year2, as explained by unmet needs in year1, when controlled by health status in year2. The two equations are estimated simultaneously, enabling to identify causalities between unmet needs, health status and health care utilization.

## Results

Results show a significant detrimental effect of unmet needs on future health status. Having given up on care in year1 appears significantly correlated with a loss in self assessed health status in year2 (O.R = 1.4). The associated impact on health consumption remains nevertheless insignificant.

## Discussion

This study brings new evidence on the impact of financial barriers in access to care on health and health consumption. It also clarifies causality pathways between health care consumption and health. We also reach methodological conclusion by showing self assessed unmet needs a convenient and appropriate tool to examine issues related to equity in access to care.
